# A pacemaker-assisted microvascular decompression for a patient with left primary facial spasm and arrhythmia: a case report

**DOI:** 10.1186/s12893-020-01025-x

**Published:** 2021-01-06

**Authors:** Yufei Liu, Jihu Yang, Xiejun Zhang, Fanfan Chen, Liwei Zhang, Guodong Huang

**Affiliations:** 1 Neurosurgical Department, The First Affiliated Hospital of Shenzhen University, Shenzhen Second People’s Hospital, Shenzhen, China; 2 Neurosurgical Department, Beijing Tiantan Hospital, Capital Medical University, No. 119, South Fourth Ring West Road, Fengtai District, Beijing, 100070 China

**Keywords:** Facial spasm, Microvascular decompression, Cardiac pacemaker, Case report

## Abstract

**Background:**

Primary facial spasm accompanied by arrhythmia is a rare clinical phenomenon and has not been reported before. We describe this phenomenon and discuss its mechanism and treatment.

**Case presentation:**

We herein present a rare case of a patient with left primary facial spasm and a third-degree atrioventricular block (III degree AVB), who was implanted with a temporary cardiac pacemaker to receive microvascular decompression (MVD) because of refusal of a permanent cardiac pacemaker. The symptoms of facial spasm disappeared after MVD. The temporary cardiac pacemaker was removed on the second day after surgery. Her ECG still showed the third-degree atrioventricular block after a follow-up period of 5 months.

**Conclusions:**

We are the first to report a patient with facial spasm and arrhythmia who was implanted with a temporary cardiac pacemaker to receive MVD. This case report demonstrated that the concomitant presence of a III degree AVB maybe not a contraindication for MVD, and the etiology of this facial spasm was the actual vascular compression of the facial nerve entry zone that was not related to the atrioventricular block.

## Background

Hemifacial spasm (HFS) is defined as unilateral, involuntary, irregular tonic or clonic contractions of the facial muscles triggered by an ipsilateral irritated cranial nerve VII. The most common etiology for primary hemifacial spasm reported in the literature is an offending artery, which compresses the facial nerve at this root entry/exit zone (REZ) [1]. Botulinum toxin therapy and MVD of the facial nerve are the main treatments for primary HFS [1]. The adverse effects of botulinum toxin include the cost of therapy and need for repeated injections, facial asymmetry, mild facial paresis, diplopia, and ptosis. The MVD has a success rate of 85.1% according to a systematic review [2]. To the best of our knowledge, there has been no previous report about the phenomenon of HFS accompanied by third-degree atrioventricular block. We herein describe this phenomenon and discuss its possible causes and treatment.

## Case presentation

### Patient information and clinical findings

**Fig. 1:**
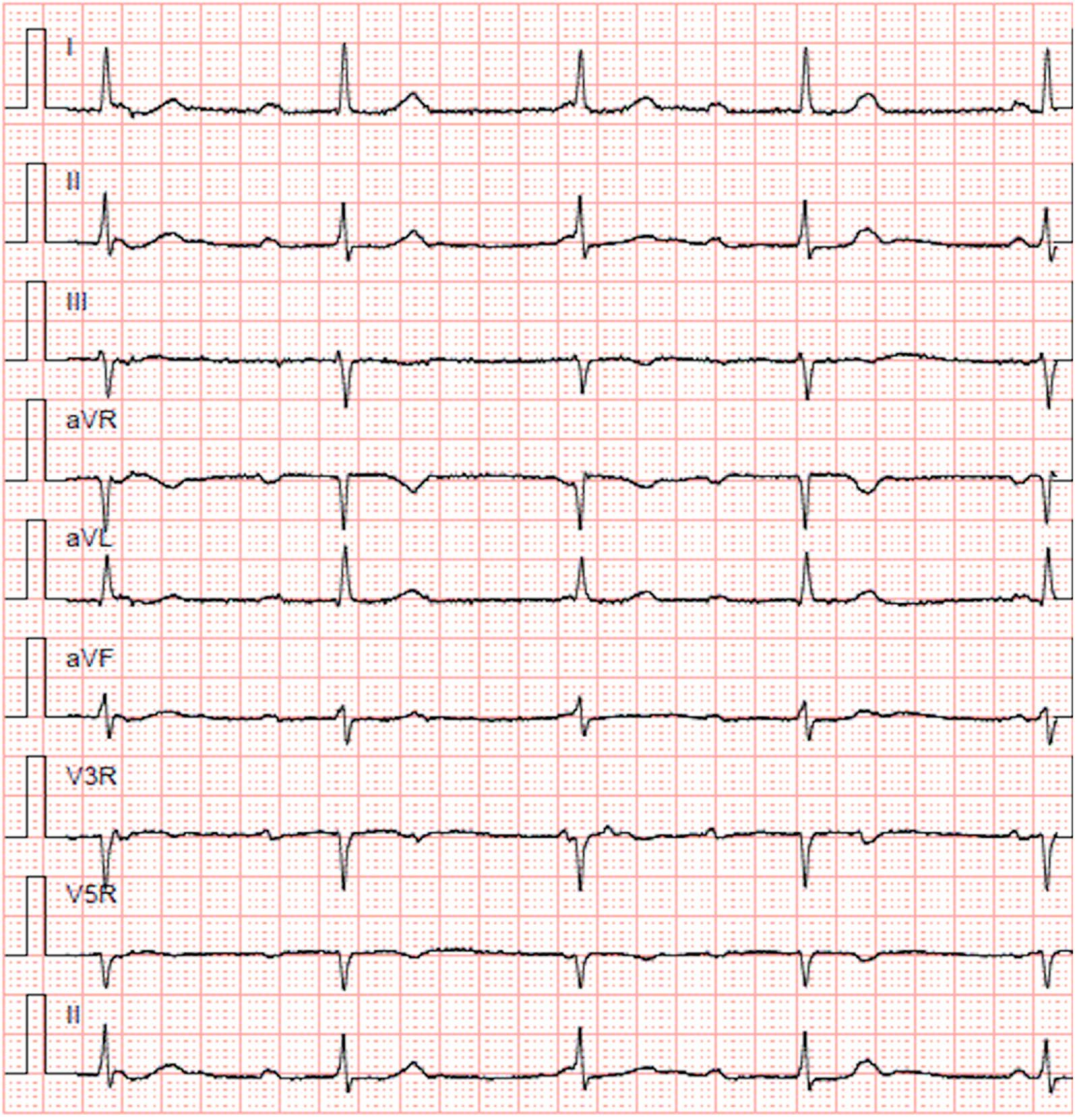
Preoperative ECG showed third-degree atrioventricular block

**Fig. 2:**
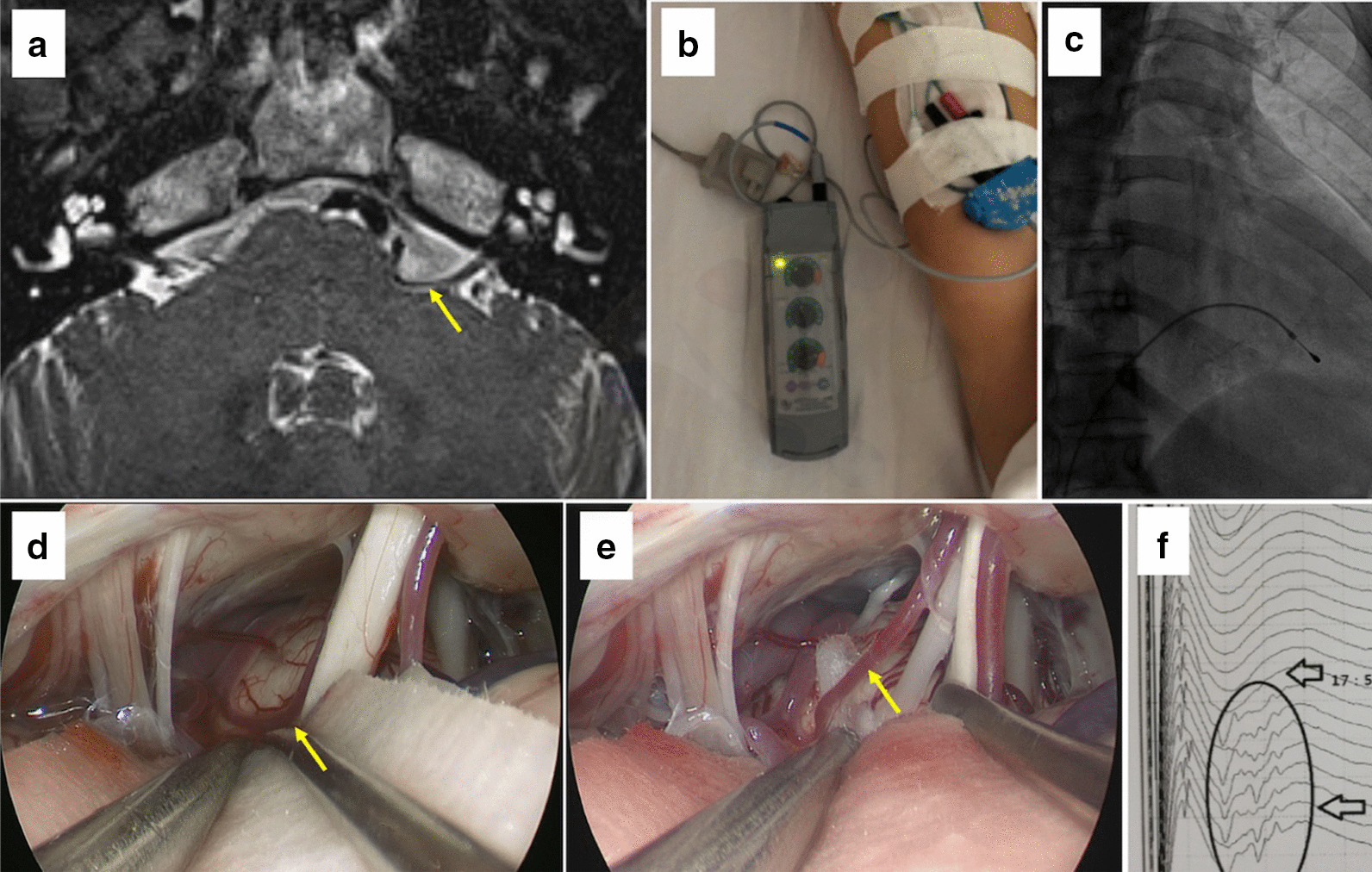
The offending artery (**a**). A temporary cardiac pacemaker was implanted (**b**, **c**). The offending artery (**d**) and completed decompression of offending vessels (**e**)

The 50-year-old female patient, with unexplained deafness on her left side for more than 10 years, complained of involuntary left HFS for 8 years. After admission, the ECG before surgery showed third-degree atrioventricular block (Fig. 1). Due to the fear of surgery, heart risk and fears related to her father’s death after the implantation of a permanent cardiac pacemaker 2 years previously, the patient previously chose botulinum toxin therapy multiple times until this therapy failed to control the spasm. On admission, she had a previous history of hypertension but no chest pain or chest tightness. Except for left deafness and left HFS, her other neurological examination was normal. MRI examination showed that bilateral vertebral arteries were located on the left side of the brain stem, and the offending artery compressed the facial nerve at this root entry/exit zone (REZ) (Fig. 2a).

### Therapeutic intervention

She agreed to be implanted with a temporary cardiac pacemaker (Fig. 2b, c), not a permanent one, to receive an MVD according to cardiac experts’ advice. The endoscopy-assisted MVD was performed successfully on 2019-11-14 under neuro-electrophysiological examination, with the heart rate set at 60 times/min (Fig. 2d, e). After the MVD, the patient’s facial spasm and abnormal muscle response (AMR) disappeared (Fig. 2f). The pacing wire was removed on the second day after surgery. The patient recovered and was discharged from the hospital.

### Follow-up and outcomes

Her ECG 5 months after surgery still showed the third-degree atrioventricular block. The patient had returned to her daily life and was asymptomatic.

## Discussion and conclusion

The etiology of the primary HFS has been generally agreed to be the result of vascular compression of the facial nerve. Botulinum toxin therapy, with the advantages of minimal side effects and ease of therapy, is widely considered as a therapeutic choice for the patient who is afraid of craniotomy. Surgical MVD is suitable for all those patients with HFS who are medically fit and willing to undergo MVD that could offer the best prospect of a permanent cure, especially if there is any MRI demonstrable pathology that is amenable to surgical removal. AMR could be an excellent tool for successful MVD in patients with primary HFS. The endoscope-assisted retrosigmoid approach technique provides an optimal visualization of the neurovascular conflict, allowing an accurate decompression of the cranial nerve VII with low complication rates.

In this case, because she feared the surgical and heart risk due to her father’s death after being implanted with a permanent cardiac pacemaker 2 years previously, she agreed to implant a temporary cardiac pacemaker for MVD according to cardiac expert’s advice. Implantation of a temporary cardiac pacemaker to ensure the patient gets through the operation period safely may be a good choice for these cardiac patients [3]. Temporary cardiac pacing may be considered in some patients who have the opportunity of resolution of atrioventricular block.

There are reports showing the relationship between HFS and heart rate. A mechanical compression of the facial nerve and fluctuations in heart rates may contribute to the occurrence of HFS [4]. Zhang et al. [5] maintained that most neurogenic sinus bradycardia occurred in right-sided HFS, and decompression at the REZ of the facial and vagus nerves and/or ventrolateral medulla oblongata simultaneously was an effective treatment for HFS. We agree that fluctuations in heart rate and compression of the facial nerve causing facial nerve hyperexcitability may be the mechanisms of HFS. The HFS in this case disappeared after the MVD, but the atrioventricular block had not disappeared at the 5-month follow-up, indicating that the etiology of this facial spasm was the actual vascular compression of the facial nerve entry zone that was not related to the atrioventricular block. More cases are needed to confirm the relationship between arrhythmia and facial spasm. In addition, we do not know the cause of left deafness. The cause may be the long-term compression of REZ by the artery, resulting in the auditory nerve being pulled.

In conclusion, this case report demonstrated that the concomitant presence of a III degree AVB maybe not a contraindication for MVD, and the etiology of this facial spasm was the actual vascular compression of the facial nerve entry zone that was not related to the atrioventricular block.

### Patient perspective

Implantation of a temporary cardiac pacemaker to ensure the patients get through the MVD operation period safely may be a good choice for patients like me with the arrhythmia.

## Data Availability

Not applicable.

## Abbreviations

MVD: Microvascular decompression
ECG: Electrocardiography
HFS: Hemifacial spasm
REZ: Root entry/exit zone
AMR: Abnormal muscle response

## References

[1] Rosenstengel C, Matthes M, Baldauf J (2012). Hemifacial spasm: conservative and surgical treatment options. Dtsch Arztebl Int.

[2] Xia L, Zhong J, Zhu J (2015). Delayed relief of hemifacial spasm after microvascular decompression. J Craniofac Surg.

[3] Csontos C, Bogar L, Melczer L (2003). Temporary pacemakers for non-cardiac surgery. Eur J Anaesthesiol.

[4] Hamasaki T, Morioka M, Fujiwara K (2018). Is hemifacial spasm affected by changes in the heart rate? A study using heart rate variability analysis. Clin Neurophysiol.

[5] Zhang G, Chen G, Zuo H (2009). First description of neurogenic sinus bradycardia in idiopathic hemifacial spasm. Surg Neurol.

